# Bone Mineral Density, Body Composition, and Mineral Homeostasis Over 24 Months in Urban South African Women With HIV Exposed to Antiretroviral Therapy

**DOI:** 10.1002/jbm4.10343

**Published:** 2020-03-18

**Authors:** Matthew M Hamill, John M Pettifor, Kate A Ward, Shane A Norris, Ann Prentice

**Affiliations:** ^1^ Medical Research Council Elsie Widdowson Laboratory Cambridge UK; ^2^ South African Medical Research Council/University of Witwatersrand Developmental Pathways for Health Research Unit, Faculty of Health Sciences University of Witwatersrand Johannesburg South Africa; ^3^ Division of Infectious Diseases, Bayview Medical Center Johns Hopkins University School of Medicine Baltimore MD USA

**Keywords:** AFRICA, ANTIRETROVIRAL THERAPY, BONE HEALTH, HIV, PREMENOPAUSAL WOMEN

## Abstract

Human immunodeficiency virus‐ (HIV‐) infection and antiretroviral therapy (ART) exposure are associated with bone loss. African data are limited despite the region's HIV burden. Of 247 ART‐naïve, premenopausal, urban, black African women aged 33.9 ± 6.6 years from Soweto, South Africa, measured at baseline, 110 underwent anthropometry, DXA, and blood and urine collections at 12 and 24 months; 39 were HIV‐negative (Nref), 28 were people with HIV (PWH) not ART‐exposed for the duration of the study (ART‐N), and 43 were PWH who were ART‐exposed within the first 12 months (ART‐Y). At baseline, the ART‐Y group had lower BMI and fat mass than the Nref group. Within 12 months of ART initiation, areal bone mineral density (aBMD) had decreased at the lumbar spine and at the whole body less head, despite increased weight, and hip aBMD had not increased in line with the Nref group. There was no evidence of further bone changes between 12 and 24 months. By 24 months, the ART‐Y women had gained weight and fat mass, but remained lighter with less fat than the Nref women. ART initiation normalized the low serum albumin of the ART‐Y group at baseline, but was associated with elevated bone turnover markers at 12 and 24 months. Vitamin D status and renal phosphate handling were normal. ART‐N had similar aBMD and other characteristics to the Nref group throughout, except unlike the Nref group, weight and fat mass did not increase and serum albumin decreased. This study in African women of childbearing age demonstrated that the bone loss that had occurred in these PWH after ART initiation did not continue after 12 months and that bone loss did not occur in ART‐unexposed PWH over 2 years. At 24 months, despite gains in weight and fat mass, ART‐exposed women remained lighter, with lower aBMD, fat mass, and higher bone turnover than women without HIV. More studies are required to establish if the bone loss and fat gain reverse, stabilize, or continue with further ART exposure, particularly during and after menopause. © 2020 The Authors. *JBMR Plus* published by Wiley Periodicals, Inc. on behalf of American Society for Bone and Mineral Research.

## Introduction

Despite the ongoing high burden of human immunodeficiency virus (HIV) in sub‐Saharan Africa, the availability of effective combination antiretroviral therapy (ART) has resulted in dramatic survival gains,^1^ which are anticipated to continue.^2^ With improved survival there has been a greater focus on HIV as a chronic disease of aging and on HIV‐associated noncommunicable diseases, including bone loss and fractures.^3^ Although data on female participants^4^, ^5^, ^6^, ^7^, ^8^ have started to emerge from resource‐limited settings where osteoporotic fractures are predicted to increase,^9^ most studies to date have predominantly been from high‐income settings and focused on males, who generally have the lowest lifetime risk of fragility fracture.

There remains uncertainty about the underlying mechanisms and the relative contributions of HIV infection, ART, and lifestyle factors on bone mineral loss. However, the balance of evidence suggests that ART exposure is a leading contributor.^10^, ^11^ Studies have demonstrated a 2% to 6% reduction in areal bone mineral density (aBMD) following initiation of ART, occurring generally over the first 2 years. Most published studies, from high‐income countries, report no further reduction in aBMD after 2 years.^12^, ^13^, ^14^ In an early seminal meta‐analysis,^12^ people with HIV (PWH) were three times more likely to have low aBMD than controls. In a 2018 meta‐analysis, PWH and ART‐exposed had an OR of osteoporosis/osteopenia of 2.8 and 3.4 at the lumbar spine and total hip (TH) compared with PWH but ART‐naïve.^15^ The ART agent, tenofovir disoproxil fumarate (TDF) is particularly associated with bone loss both in people with^12^, ^16^, ^17^ and without HIV.^18^ The underlying mechanism has not been firmly established; diverse explanations have been proffered, including renal phosphate loss, immune reconstitution, inadequate vitamin D status, and consequences of macrophage activation.^19^, ^20^, ^21^

We have previously demonstrated in a cross‐sectional analysis of a cohort of urban, premenopausal South African women, comparing PWH but ART‐naïve with those who were HIV‐negative, that there were no significant differences in aBMD related to HIV status. This was despite women with HIV with low CD_4_ counts being less adipose than those with preserved CD_4_ counts or HIV‐negative women.^4^ In a follow‐up study at 12 months,^5^ there were significant decreases in aBMD despite significant increases in weight and fat mass. These effects were associated with increased bone turnover markers, but there were no differences or changes in vitamin D status, serum phosphate concentration, or renal phosphate handling.^5^

We hypothesized that these African women with HIV and ART exposed would continue to lose bone mineral after 12 months of therapy. The aim of the study presented here was to follow this cohort of women to 24 months to investigate the effects of continued HIV infection and ART exposure on BMD and to consider the influence of potential modulators by measuring body composition, vitamin D status, and markers of mineral metabolism and inflammation.

## Participants and Methods

### Study design

The study was designed as a 24‐month longitudinal investigation of urban, black South African premenopausal women with and without HIV infection. Participants attended for study visits between February and July 2010 at the SAMRC/Wits Developmental Pathways for Health Research Unit (DPHRU) in Soweto, Johannesburg, South Africa, at baseline and at 12‐ and 24‐months postbaseline. At each visit, anthropometry and DXA scans were performed and biological samples collected. This report focuses on those participants who attended at all three time points and whose data were not excluded because of an intervening pregnancy/lactation or, in the case of the HIV‐negative women, seroconversion.

**Figure 1:**
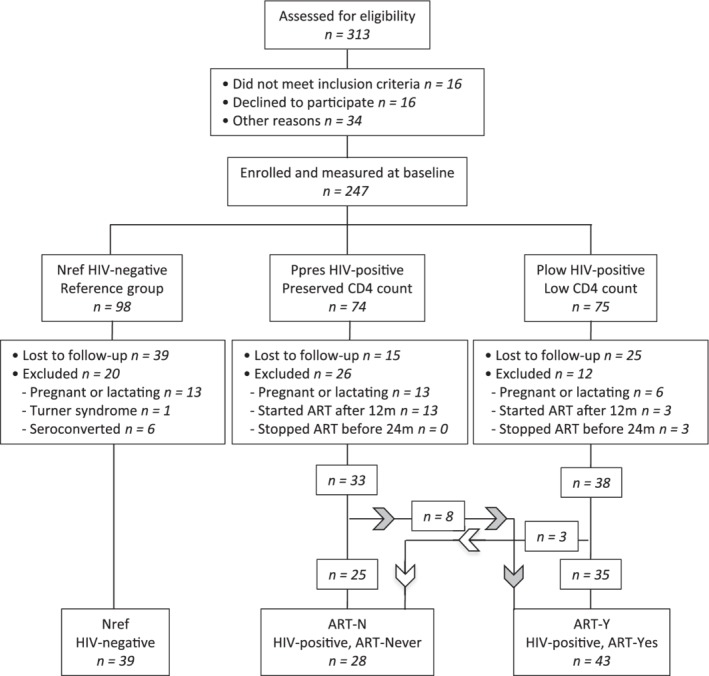
Progress of participants through the study to 24 months. Women with HIV who started ART after 12 months or stopped before 24 months were excluded from data analysis. Nref = HIV‐negative reference women; Ppres = women with HIV with preserved CD4 counts at baseline; Plow = women with HIV with low CD4 counts at baseline eligible to start ART; ART‐N = women with HIV from Ppres and Plow who did not start ART by 24 months; ART‐Y = women with HIV from Ppres and Plow who started ART before 12 months and remained on ART to 24 months.

Details of the study protocol, inclusion and exclusion criteria, baseline characteristics, and changes to 12 months have been described in full elsewhere.^4^, ^5^, ^22^ Briefly, 247 women were recruited into three groups of approximately equal size from clinics in Soweto between February and July 2010. All were premenopausal and not pregnant/lactating at the time of enrollment. The groups at recruitment were: (i) women without HIV to serve as the reference group (Negative‐reference: Nref, *n* = 98); (ii) women with HIV and preserved CD_4_ counts (≥ 350 × 10^6^ cells/L) and anticipated not to require ART initiation for at least 12 months (Positive‐preserved: Ppres, *n* = 74); and (iii) women with HIV and low CD_4_ counts (≤200 × 10^6^ cells/L) eligible to commence ART (Positive‐low: Plow *n* = 75). The criteria used for commencing ART were those in place nationally at the time of commencement of the study. At the 12‐ and 24‐month visits, Nref participants were offered repeat HIV‐antibody testing using the Alere Determine Rapid HIV‐Antibody Test (Alere San Diego, Inc., San Diego, CA, USA). Those who had a reactive HIV test were referred to a local primary health care facility for confirmatory testing, CD_4_ count, and consideration of ART (Fig. 1).

By 12 months, most of the Plow group had initiated ART, as had many of the Ppres group because of revised initiation guidelines in South Africa. To explore the primary research question about the effect of ART for longer than 12 months, the data from women with HIV were grouped into those who had initiated ART before 12 months and continued to 24 months (ART‐Y) and those unexposed to ART throughout (ART‐N). Those Nref women measured at 12 and 24 months and still fulfilling the eligibility criteria provided normative data.

The University of the Witwatersrand Human Research Ethics Committee (HREC number: M101525) and the Gauteng Department of Health, South Africa, approved the study. All participants provided informed written consent prior to enrollment. Those with HIV continued to attend their usual primary health care facilities for ongoing clinical care.

### Anthropometry

Height was measured to the nearest 0.1 cm using a permanent wall‐mounted stadiometer (Holtain, Crosswell, UK). Weight was measured to the nearest 0.1 kg using an electronic digital scale (Tanita TBF‐410 MA Body Composition Analyzer, Tanita Corporation of America, Inc., IL, USA) with participants wearing light clothing. BMI was calculated as weight in kilograms divided by the square of height in meters (kg/m^2^). Waist and hip circumferences were measured to the nearest 0.1 cm using a nonstretchable plasticized tape measure.

### Bone mineral density and body composition by DXA

DXA was performed using an Hologic QDR 4500A DXA [Model: Discovery W (S/N 71201), software version 12.5:7; Hologic, Inc., Waltham, MA, USA] to obtain measures of aBMD, BMC, and bone area (BA). Scans of the whole body (WB), lumbar spine L1 to L4 (LS), TH, and femoral neck (FN) were conducted with participants wearing light clothing, and performed using the automatic scan mode. WB was analyzed as “whole body less head” (WBLH) because of the high proportion of women with nonremovable hair weaves that compromise DXA assessment of head aBMD. For each individual, their follow‐up scans were compared with baseline to ensure consistent placement of regions of interest. The extent to which individuals experienced aBMD loss in excess of the least significant change was determined using the conventional DXA 0.03 g/cm^2^ threshold, which is based on a notional instrument precision of 1%.^23^

### Laboratory measures

Full details of the blood and urine collection, processing, and analytical procedures, including assay and manufacturer information are described in detail elsewhere.^4^, ^5^ In brief, blood was collected in the morning after an overnight fast and processed as EDTA plasma for PTH analysis and as serum for other analytes relating to calcium, phosphorus, vitamin D metabolism [calcium, phosphate, magnesium, albumin, 25(OH)D], bone turnover (total alkaline phosphatase [TALP], P1NP, and serum collagen type 1 crosslinked β‐C‐telopeptide [β‐CTx]), and inflammation (C‐reactive protein [CRP], ferritin). All plasma and serum samples were stored frozen, initially at –20°C and subsequently at –80°C. Urine was collected into a sterile container at the second void of the day after an overnight fast, acidified 10 μL/mL with concentrated hydrochloric acid, and stored at –20°C (see supplementary data for full description).

### Statistical methods

Data were analyzed using DataDesk 6.3.1 (Data Description, Inc., Ithaca, NY, USA). Summary statistics are presented as mean ± SD for normally distributed data or median (25th percentile, 75th percentile [IQR]) for skewed distributions. Based on findings from the baseline study, fat mass‐to‐lean mass^2^ (fat:lean^2^) was used to describe body composition.^4^ All continuous variables were transformed to natural logarithms prior to data analysis allowing differences between groups and between timepoints to be expressed as sympercents ([difference/mean] × 100)^24^ and for positively skewed distributions to be normalized. Summary sympercent data are presented as percentage mean difference ± SE.

Full details of the statistical approach have been described previously.^5^ Group and timepoint differences were investigated using ANOVA, utilizing the linear model software in DataDesk with Scheffé post hoc tests. Repeat‐measures hierarchical models were constructed to evaluate and compare the within‐individual changes in each variable over time between the three groups. The results of two models are presented: (i) two‐timepoint analysis (12/24 months) to focus on changes after 12 months; and (ii) three‐timepoint analysis (0/12/24 months) to consider whether the changes associated with ART initiation were also seen in this subset of the original cohort. In addition to timepoint, these models included individual identifier (nested by group), group (Nref/ART‐N/ART‐Y), and a group‐by‐timepoint interaction term. The bone data were also modeled with BA and body weight as explanatory variables, to adjust for changes in bone and body size.^5^, ^25^ No additional adjustment for covariates such as lifestyle factors was made because they did not differ significantly between the groups.^5^

## Results

The progress of participants through the study and the reasons for loss to follow‐up are illustrated in Fig. 1. Of the 247 women measured at baseline, 79 were not available at 12 and/or 24 months, usually because they had moved away. Additionally, data from 32 women were excluded because of pregnancy/lactation in the interim period, 1 because of a diagnosis of Turner syndrome, and 6 because of newly acquired HIV infection in the Nref group. In addition, data from 16 women with HIV who started ART after the 12‐month timepoint and 3 who had initiated ART but stopped by 24 months were excluded. Thus, the analyzed dataset comprised 39 Nref, 28 ART‐N with HIV and ART‐unexposed, and 43 ART‐Y with HIV and had initiated ART before 12 months and continued ART to 24 months.

The majority of women in ART‐Y were prescribed contemporary standard‐of‐care first‐line ART medication. More than 90% of ART‐Y were taking TDF, 86% lamivudine, and 91% a nonnucleoside reverse‐transcriptase inhibitor, efavirenz (79%) or nevirapine (12%), as the third agent. Only three women switched during the study to second‐line therapy with lamivudine/zidovudine (*n* = 2) or raltegravir/lopinavir/ritonavir (*n* = 1). The median duration of ART exposure in ART‐Y was 685 [591,742] days by 24 months. Among women with HIV the CD_4_ count (counts × 10^6^/L) in ART‐Y was lower at baseline than in ART‐N (median IQR = 187 [101,230] versus 430 [343,477], *p* < .0001). There were progressive increases in CD_4_ count among ART‐Y by 12 and 24 months (267 [179,350] and 446 [322,520], respectively), but no significant change among ART‐N (466 [350,492] and 477 [389,554], respectively). No minimal trauma fractures were reported during the 2 years of the study, 1 woman each in ART‐N and ART‐Y had suffered an appendicular fracture caused by a traumatic injury. No participant reported using a vitamin D supplement or a multimicronutrient supplement containing vitamin D. Two participants (Nref = 1, ART‐Y = 1) reported using a supplement containing calcium.

**Figure 2:**
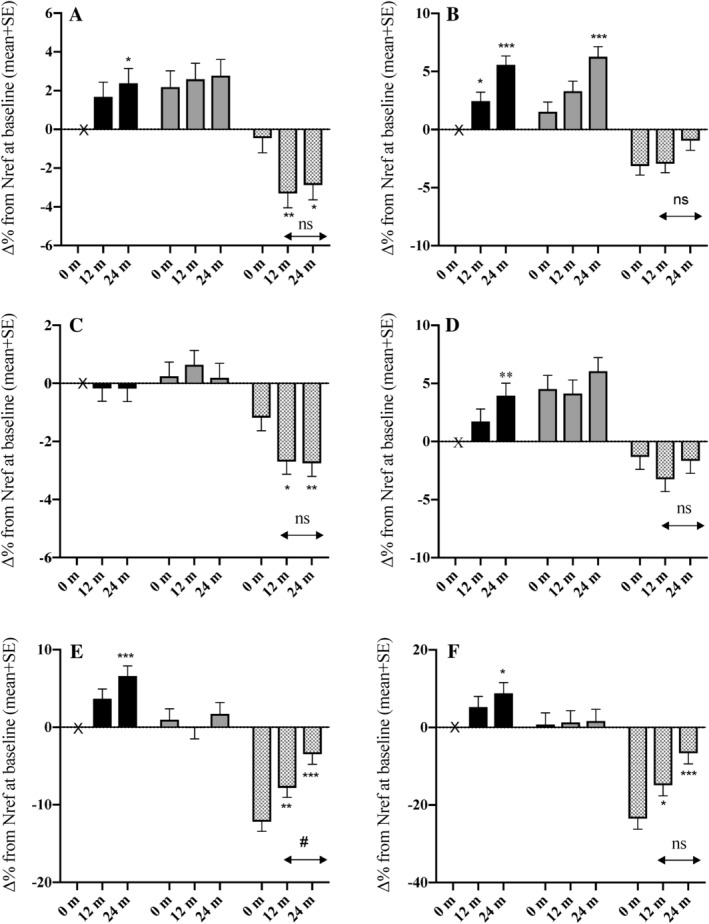
Differences between groups in percentage change in BMI, body composition, and areal bone mineral density (aBMD) by 12 and 24 months. (*A*) lumbar spine aBMD, (*B*) total hip aBMD, (*C*) whole body less head aBMD, (*D*) femoral neck aBMD, (*E*) BMI, (*F*) fat:lean^2^ ratio. Data for each variable at 0 months, 12 months, and 24 months are the percentage difference (∆%) relative to the value of Nref at baseline: black bars = Nref (HIV‐negative women, *n* = 39), gray bars = ART‐N (women with HIV not ART‐exposed by 24 months, *n* = 28), hatched bars = ART‐Y (women with HIV who initiated ART before 12 months and continued to 24 months, *n* = 43). Data were obtained from Scheffé post hoc tests from hierarchical linear models of the variable in natural logarithms with group (Nref/ART‐N/ART‐Y), timepoint (0/12/24 months), ID (nested within group), and a group‐by‐timepoint interaction. Error bars are SE. Significance of change from baseline within each group: *p ≤ 0.05, **p ≤ 0.01, ***p ≤ 0.001. Significance of change within each group between 12 and 24 months: ns = not significant (*p* > 0.05), #*p* ≤ 0.05. Significance of differences between groups at each timepoint are given in Supplementary Table S1.

Table 1 provides details of the aBMD, anthropometry, and body composition of the three groups at 12 months and 24 months and gives, for each variable, the statistical significance from the two‐timepoint model of the between‐group differences at each timepoint, the within‐individual changes in each group between 12 and 24 months, and the group‐by‐timepoint interaction terms that indicate whether the time effects differed significantly between groups. The percentage differences in these variables from the Nref group at 12 and 24 months for the ART‐N and ART‐Y groups are provided in Table 2. To place these results in context with the changes that occurred in the first 12 months, Fig. 2 A, B, C, D, E and F illustrates the percentage differences in aBMD, BMI, and fat/lean^2^ in each group at 0, 12 months, and 24 months expressed relative to the Nref group at baseline. The summary data, changes from baseline, and differences from the Nref group at baseline, 12 months, and 24 months are given in Supplementary Tables S1 to S3.

**Table 1:** Anthropometry and Bone Mineral Densities by ART Status in Women Participating 12 and 24 Months

	Nref		ART‐N		ART‐Y		Interaction*
	12 months	24 months	12 months	24 months	12 months	24 months	*p*
*Bone mineral density (aBMD g/cm* ^*2*^ *)*							
Lumbar spine	1.031 ± 0.119	1.039 ± 0.122	1.040 ± 0.116	1.043 ± 0.120	0.983 ± 0.124^**a,d**^	0.990 ± 0.127^**a,d**^	0.93
Total hip	1.041 ± 0.142	1.073 ± 0.139^**h**^	1.051 ± 0.146	1.079 ± 0.147^**i**^	0.985 ± 0.126^**a,d**^	1.002 ± 0.124^**a,d,i**^	0.61
Femoral neck	0.923 ± 0.137	0.960 ± 0.152^**h**^	0.962 ± 0.144^**c**^	0.984 ± 0.174	0.892 ± 0.132^**c,d**^	0.909 ± 0.144^**a,d**^	0.40
WBLH	0.965 ± 0.081	0.966 ± 0.084	0.973 ± 0.077	0.969 ± 0.836	0.942 ± 0.076^**a,d**^	0.940 ± 0.078^**a,d**^	0.79
*Anthropometry*							
Weight (kg)	74.4 ± 15.8	76.4 ± 15.8	74.0 ± 13.9	73.0 ± 13.9	68.4 ± 16.7^**a,d**^	69.5 ± 15.6^**a,d,g**^	0.13
BMI (kg/m^2^)	30.2 ± 6.6	31.1 ± 6.7^i^	28.9 ± 5.2^**b**^	28.6 ± 4.7^**a**^	26.9 ± 6.0^**a,d**^	27.5 ± 5.5^**a,d,g**^	0.29
Fat mass, WBLH (kg)	29.8 ± 11.6	31.0 ± 11.4	28.8 ± 8.5	29.4 ± 8.8	24.1 ± 11.2^**a,d**^	25.8 ± 11.6^**a,d,g**^	0.07
Lean mass, WBLH (kg)	38.9 ± 4.7	39.2 ± 5.2	39.7 ± 6.4	39.8 ± 6.2	38.3 ± 5.8^**c,d**^	38.3 ± 5.3^**e**^	0.91
Fat:lean^2^ (1000*kg/kg^2^)	19.3 ± 4.9	20.0 ± 5.3	18.6 ± 4.7	18.7 ± 4.6^**c**^	16.0 ± 5.0^**a,d**^	17.2 ± 5.3^**a,d,h**^	0.13
Waist (cm)	91.9 ± 12.4	90.2 ± 18.5	92.7 ± 13.4	87.5 ± 22.5	89.2 ± 14.6^**b,d**^	86.9 ± 19.0^**b,f**^	0.74
Hip (cm)	111.6 ± 13.4	114.4 ± 13.0	110.3 ± 9.3	111.1 ± 7.9	105.3 ± 12.4^**a,d**^	106.6 ± 12.0^**a,d,i**^	0.86
Waist:hip (cm/cm)	0.82 ± 0.07	0.79 ± 0.14	0.84 ± 0.08	0.82 ± 0.09	0.84 ± 0.07^**c**^	0.81 ± 0.14^**c**^	0.96

Nref, *n* = 39, HIV‐negative; ART‐N, *n* = 28, people with HIV (PWH) not on ART 0 to 24 months; ART‐Y, *n* = 43; PWH on ART at 24 months who initiated prior to 12 months, Data are means ± SDs, aBMD data are unadjusted. Variables with missing datapoints are in the footnote to Supplementary Table S1.

Significance of differences from Scheffé post hoc tests from hierarchical linear models of the variable in natural logarithms in the two‐timepoint model with timepoint (12/24 months), group (Nref/ART‐N/ART‐Y), ID (nested within group) and a group‐by‐timepoint interaction, as follows:

between ART‐N or ART‐Y and Nref at each timepoint ^**a**^ ≤0.001, ^**b**^ ≤0.01, ^**c**^ ≤0.05;

between ART‐N and ART‐Y at each timepoint: ^**d**^ ≤0.001, ^**e**^ ≤0.01, ^**f**^ ≤0.05;

between 12 and 24 months in each group: ^**g**^ ≤0.001, ^**h**^ ≤0.01, ^**i**^ ≤0.05.

aBMD = areal bone mineral density; ART = antiretroviral therapy; ART‐N = those unexposed to ART throughout; ART‐Y = those who had initiated ART before 12 months and continued to 24 months; HIV = human immunodeficiency virus; Nref = HIV‐negative reference women; WBLH = whole body less head.

* Interaction *p* = significance of group‐by‐timepoint interaction term in the two‐timepoint model (12/24 months).

**Table 2:** Difference in Anthropometry and Bone Mineral Densities Relative to Nref in ART‐N and ART‐Y Groups at 12 and 24 Months

	ART‐N		ART‐Y	
	12 months	24 months	12 months	24 months
	%	%	%	%
*aBMD unadjusted*				
Lumbar spine	+0.9 ± 1.0	+0.4 ± 1.0	−5.0 ± 0.9^a^	−5.2 ± 0.9^a^
Total hip	+0.9 ± 0.9	+0.7 ± 1.0	−5.4 ± 0.8^a^	−6.5 ± 0.8^a^
Femoral neck	+4.0 ± 1.3^b^	+2.1 ± 1.3	−3.4 ± 1.2^c^	−5.6 ± 1.2^a^
WBLH	+0.8 ± 0.5	+0.4 ± 0.5	−2.6 ± 0.5^a^	−2.5 ± 0.5^a^
*Anthropometry*				
Weight	−0.1 ± 1.1	−1.6 ± 1.1	−9.0 ± 0.9^a^	−7.4 ± 1.0^a^
BMI	−3.7 ± 1.1^b^	−4.6 ± 1.2^a^	−11.5 ± 1.0^a^	−9.8 ± 1.1^a^
Fat mass	−1.1 ± 2.6	−4.6 ± 2.6	−23.6 ± 2.4^a^	−18.6 ± 2.4^a^
Lean mass	+1.4 ± 0.8	+1.3 ± 0.8	−1.8 ± 0.7^c^	−1.5 ± 0.7
Fat:Lean^2^	−3.9 ± 2.7	−7.2 ± 2.7^c^	−20.0 ± 2.5^a^	−15.6 ± 2.5^a^

Nref, *n* = 39, HIV‐negative; ART‐N, *n* = 28, people with HIV (PWH) not on ART 0 to 24 months; ART‐Y, *n* = 43; PWH on ART at 24 months who initiated prior to 12 months. Data are percentage mean differences relative to Nref at the same timepoint ± SEs.

Significance of differences between ART‐N or ART‐Y and Nref at each timepoint from Scheffé post hoc tests hierarchical linear models of the variable in natural logarithms in the 2‐timepoint model with timepoint (12/24 months), group (Nref/ART‐N/ART‐Y), ID (nested within group) and a group‐by‐timepoint interaction:

a ≤0.001,

b ≤0.01,

c ≤0.05.

The significance of other comparisons between groups and across time can be found in Tables 1 and supplementary tables.

aBMD = areal bone mineral density; ART = antiretroviral therapy; ART‐N = those unexposed to ART throughout; ART‐Y = those who had initiated ART before 12 months and continued to 24 months; HIV = human immunodeficiency virus; Nref = HIV‐negative reference women; WBLH = whole body less head.

The mean ages at baseline were Nref = 32.8 ± 8.8 years, ART‐N = 33.9 ± 6.0 years, and ART‐Y = 35.0 ± 5.4 years (*p* = .38); there was no difference in height between the groups. In line with the analysis of the larger cohort followed to 12 months,^5^ there were no significant differences in cross‐sectional tests between the groups at baseline in aBMD at any of the four skeletal sites, although the longitudinal models indicated a greater FN aBMD in the ART‐N group and lower TH aBMD in the ART‐Y group than in theNref group (Supplementary Table S1). Between baseline and 12 months, LS and WBLH aBMD decreased significantly in the ART‐Y group, on average by 2.9% and 1.5%, respectively, but not in the Nref and ART‐N groups (Fig. 2, Supplementary Table S2). By 12 months, there was no significant change in TH aBMD in the ART‐Y women, but there were marked increases in the Nref and ART‐N women (Fig. 2, Supplementary Tables S1 to S3). ART‐Y participants were lighter at baseline, with lower BMI, fat mass, lean mass, fat:lean^2^, hip and waist circumferences, and higher waist:hip ratio than the Nref and ART‐N participants. By 12 months, their body weight, BMI, and several other anthropometric variables had increased, but remained lower than both the Nref and ART‐N groups (Supplementary Table S1, Fig. 2). Significant group‐by‐timepoint interaction terms in the three‐timepoint model confirmed that the patterns of within‐individual change over time differed between the groups (Supplementary Table S1).

Between 12 and 24 months (Table 1), there were no significant changes in aBMD at any site except for the TH, where an increase was observed in all groups. The lack of significant group‐by‐time interaction terms in the two‐timepoint model confirmed that the group dependent changes were confined to the first 12 months. However, the trends in the Nref group for increasing aBMD at the LS, TH, and FN continued, and by 24 months, the increases from baseline were significant (Fig. 2, Supplementary Tables S1 and S3). In consequence, the differences between the ART‐Y and Nref groups at 24 months were increased such that the ART‐Y group had lower LS, TH, FN, and WBLH aBMD compared with the Nref group on average by 5.3%, 6.5%, 5.6%, and 2.6%, respectively (Table 2). In addition, on an individual basis, a greater proportion of women in the ART‐Y group at 24 months had lower aBMD than at baseline by >0.03 g/cm^2^, most particularly at the LS [Nref = 1/39 (2.6%); ART‐N = 3/28 (11%); ART‐Y 22/43 (51%) *p* ≤ .0001], the greatest loss occurring before 12 months.

Between 12 and 24 months ART‐Y women continued to gain weight, BMI, and adiposity, as shown by increases in fat mass, fat:lean^2^ ratio, and hip circumference, but there were few significant changes in anthropometry in the Nref and ART‐N women (Table 1). However, when considered over the 24 months of the study, the Nref women had also increased significantly in weight, BMI, and adiposity compared with baseline, but the ART‐N women had not (Fig. 1, Supplementary Tables S1 and S3). Size adjustment, to make allowance for these group differences in change in weight and body composition on BMD, generally diminished the magnitude and statistical significance of the aBMD differences in ART‐Y and ART‐N relative to Nref participants, but did not alter the overall pattern (Supplementary Table S3).

**Figure 3:**
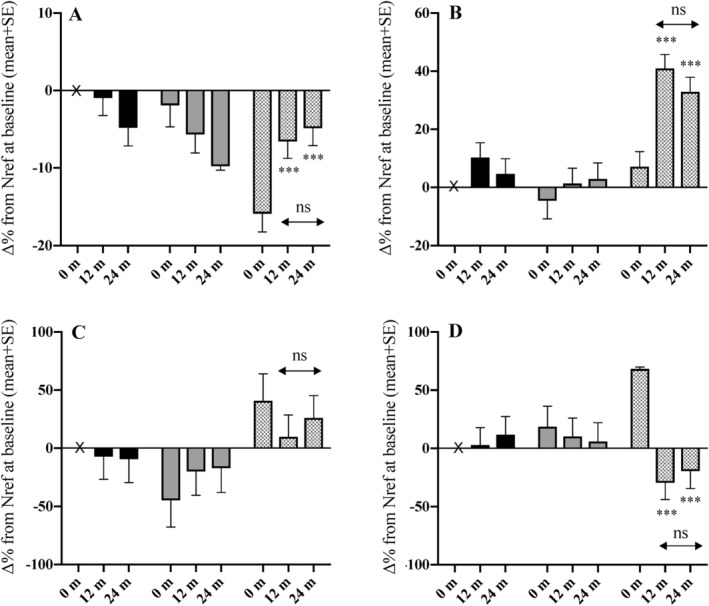
Differences between groups in percentage change in serum albumin, total alkaline phosphatase, C‐reactive protein, and ferritin by 12 and 24 months. (*A*) albumin, (*B*) total alkaline phosphatase, (*C*) C‐reactive protein, (*D*) ferritin. Data for each variable at 0 months, 12 months, and 24 months are the percentage difference (∆%) relative to the value of Nref at baseline: black bars = Nref (HIV‐negative women, *n* = 39), gray bars = ART‐N (women with HIV not ART exposed by 24 months, *n* = 28), hatched bars = ART‐Y (women with HIV who initiated ART before 12 months and continued to 24 months, *n* = 43). Data were obtained from Scheffé post hoc tests from hierarchical linear models of the variable in natural logarithms with group (Nref/ART‐N/ART‐Y), timepoint (0/12/24 months), ID (nested within group), and a group‐by‐timepoint interaction. Error bars are SE. Significance of change from baseline within each group: * *p* ≤ 0.05, ** *p* ≤ 0.01, *** *p* ≤ 0.001. Significance of change within each group between 12 and 24 months: ns = not significant (*p* > .05). Significance of differences between groups at each timepoint is given in Supplementary Table S4.

Table 3 presents the biochemistry data at 12 and 24 months. Figure 3 A, B, C and D illustrates the percentage differences from the Nref group at baseline in each of the three groups for albumin, TALP, CRP, and ferritin at 0, 12 months, and 24 months; the full summary data, including baseline values, are in Supplementary Table S4. At baseline, ART‐Y women had lower serum albumin and higher CRP and ferritin that improved after ART initiation, but serum phosphate and phosphate tubular maximum per volume of filtrate (TmP/GFR) were similar to Nref and ART‐N women. ART initiation was associated with higher TALP, P1NP, and β‐CTx at 12 months, which was sustained at 24 months. Serum albumin decreased over time in the ART‐N group. PTH was significantly higher in the ART‐Y than the ART‐N women, but not Nref women, at both 12 and 24 months. With the exception of albumin and urinary magnesium, there were no significant group‐by‐timepoint interactions in any of the factors measured between 12 and 24 months. Vitamin D status was generally good in all three groups, as demonstrated by mean 25(OH) D concentrations >50 nmol/L, and there were no group or time differences to suggest an influence on the differences and changes across time in aBMD.

**Table 3:** Biochemistry by ART Status in Women Participating at 12 and 24 Months

	Nref		ART‐N		ART‐Y		Interaction*
	12 months	24 months	12 months	24 months	12 months	24 months	*p*
*Serum*							
25 (OH)D (nmol/L)	64.9 ± 16.4	63.8 ± 19.2	69.0 ± 18.9	74.3 ± 14.5^**b**^	60.3 ± 20.2^**e**^	67.4 ± 18.1^**f,h**^	0.06
Phosphate (mmol/L)	1.15 ± 0.20	1.01 ± 0.15^**g**^	1.15 ± 0.19	1.03 ± 0.18^**h**^	1.21 ± 0.24	1.03 ± 0.14^**g**^	0.55
Calcium_corr_ (mmol/L)	2.48 ± 0.09	2.45 ± 0.10	2.48 ± 0.09	2.46 ± 0.09	2.47 ± 0.11	2.44 ± 0.09	0.94
Magnesium (mmol/L)	0.81 ± 0.07	0.80 ± 0.08	0.79 ± 0.07	0.77 ± 0.07^**c**^	0.80 ± 0.05	0.79 ± 0.07	0.70
Albumin (g/L)	40.6 ± 3.3	38.9 ± 2.8	39.0 ± 4.1^**c**^	37.7 ± 4.3^**c,i**^	38.7 ± 4.7^**b**^	38.9 ± 3.5^**e**^	0.02
Creatinine# (μmol/L)	68.5 ± 8.7	66.3 ± 10.7	62.7 ± 9.0^**b**^	58.7 ± 7.4^**a,i**^	64.0 ± 11.4^**b**^	63.5 ± 11.6^**d**^	0.07
eGFR (mL/min/1.73m^2^)	100.8 ± 14.1	102.3 ± 15.5	107.5 ± 13.2^**b**^	111.6 ± 10.3^**a,i**^	105.3 ± 14.2	104.2 ± 14.0^**d**^	0.10
TALP^¶^ (U/L)	52.8[42.9,61.0]	51.1[40.8,60.0]	45.7[40.2,59.5]	47.5[38.1,58.9]	79.3[57.7,89.3]^**a,d**^	67.3[54.1,83.8]^**a,d**^	0.32
P1NP^¶^ (μg/L)	46.1[35.8,61.0]	46.4[36.6,62.1]	50.8[37.5,71.5]	49.0[36.2,57.8]	75.4[58.9,102.3]^**a,d**^	67.2[46.9,86.7]^**a,e,i**^	0.14
β‐CTX^¶^ (ng/L)	109[69,189]	151[109,263]^**h**^	131[86,233]	176[115,252]^**i**^	183[120,256]^**a,e**^	219[131,310]	0.09
PTH^¶^ (ng/L)	23.3[15.2,31.1]	37.9[30.2,46.5]^**g**^	21.1[14.0,27.4]	27.6[20.7,38.8]^**c,h**^	23.0[16.9,33.9]^**f**^	40.6[29.4,52.5]^**e,g**^	0.58
CRP^¶^ (mg/L)	4.2[3.1,7.4]	4.1[3.1,5.0]	4.4[3.7,5.9]	4.2[3.7,5.4]	4.3[3.5,15.3]	5.1[3.8,13.2]^**f**^	0.70
Ferritin^¶^ (μg/L)	32.3[13.1,80.0]	44.3[13.6,77.4]	42.2[11.8,59.3]	38.5[16.8,63.9]	16.7[10.7,42.4]^**b,e**^	25.4[15.4,30.2]^**c**^	0.69
*Urine*							
Phosphate:Cre	1.00 ± 0.53	0.99 ± 0.62	1.28 ± 0.69	1.18 ± 0.64^**c**^	1.41 ± 0.64	1.28 ± 0.69^**b**^	0.49
Calcium:Cre^¶^	0.08[0.03,0.15]	0.06[0.03,0.15]	0.08[0.03,0.13]	0.11[0.06,0.12]	0.07[0.03,0.18]	0.08[0.02,0.19]	0.19
Magnesium:Cre	0.17 ± 0.08	0.15 ± 0.08	0.15 ± 0.07	0.15 ± 0.04	0.23 ± 0.12^**e**^	0.19 ± 0.10	0.04
TmP/GFR (mmol/L)	1.26 ± 0.32	1.08 ± 0.26^**h**^	1.20 ± 0.26	1.10 ± 0.27	1.24 ± 0.33	1.03 ± 0.21^**g**^	0.47

Nref, *n* = 39, HIV‐negative; ART‐N, *n* = 28, people with HIV (PWH) not on ART 0 to 24 months; ART‐Y, *n* = 43; PWH on ART at 24 months who initiated prior to 12 months. Data are means ± SDs for normal distributions, for those with positive skew (¶) are median [25,75 percentile]. *Interaction *p* = significance of group‐by‐timepoint interaction term in the two‐timepoint model (12/24 months). #Serum creatinine values, assayed using the Jaffe method, were corrected to provide traceability to the reference method using the compensation equation: compensated creatinine (μmol/L) = (measured value‐26.8) × 1.168 μmol/L. Variables with missing datapoints are in the footnote to Supplementary Table S4.

Significance of differences from Scheffé post hoc tests from hierarchical linear models of the variable in natural logarithms in the three‐timepoint model with timepoint (0/12/24 months), group (Nref/ART‐N/ART‐Y), ID (nested within group), and a group‐by‐timepoint interaction, as follows:

between ART‐N or ART‐Y and Nref at each timepoint ^**a**^ ≤0.001, ^**b**^ ≤0.01, ^**c**^ ≤0.05.

between ART‐N and ART‐Y at each timepoint: ^**d**^ ≤0.001, ^**e**^ ≤0.01, ^**f**^ ≤0.05;

between 12 and 24 months in each group: ^**g**^ ≤0.001, ^**h**^ ≤0.01, ^**i**^ ≤0.05.

25(OH)D = 25‐hydroxyvitamin D; aBMD = areal bone mineral density; ART = antiretroviral therapy; ART‐N = those unexposed to ART throughout; ART‐Y = those who had initiated ART before 12 months and continued to 24 months; CRP = C‐reactive protein; Cre = urine creatinine used to develop urine mineral ratios in mmol/mmol; eGFR = estimated glomerular filtration rate using the CKD‐EPI formula with no black factor; HIV = human immunodeficiency virus; Nref = HIV‐negative reference women; TALP = total alkaline phosphatase; P1NP = serum type 1 procollagen N‐terminal; β‐CTX = serum collagen type 1 cross‐linked β‐C‐telopeptide; TmP = tubular maximum reabsorption rate of phosphate.

## Discussion

This is the first prospective cohort study in African women comparing DXA‐defined changes in BMD, body composition, vitamin D status, and biochemistry over 24 months in ART‐naïve and newly ART‐initiated women with HIV with those without HIV. The inclusion of a group with HIV who were ART‐unexposed is novel and unlikely to be repeated in future studies given the moves toward earlier ART initiation.^26^

These data suggest that in South African women, HIV‐positivity has no measurable negative effects on bone mineral status over a 24‐month period, unlike the conclusions reported in earlier studies.^27^, ^28^ However, exposure to TDF‐containing ART was associated with loss of bone mineral and increased bone turnover in the first 12 months that stabilized by 24 months as in other populations studied^11^, ^12^ but not in all populations.^14^ Although the duration of HIV infection prior to participation in the study was unknown, the range of baseline CD_4_ counts across the two groups of PWH suggests that the women had been infected for different lengths of time. Baseline analysis did not reveal significant differences in aBMD between women with and without infection or between those with HIV with low and preserved CD_4_ counts. This suggests that the observed reductions in aBMD observed in the ART‐Y group were related to ART‐exposure and not primarily driven by the presence of HIV.

At 24 months, aBMD in the ART‐Y group was significantly lower than the Nref group at all sites by 2% to 7%, even after allowing for differences in weight and weight gain, with higher bone turnover indices (in our previous report we showed that the elevation in TALP in the ART‐Y group at 12 months was matched by higher bone‐specific ALP activity, indicating a bone origin rather than liver origin).^5^ There was no evidence of bone loss in the ART‐N women over 24 months and the aBMD of these women—with HIV, a preserved CD4 count, and ART‐unexposed—remained comparable to the Nref women despite not experiencing the same increase in weight. These results suggest that the overall bone health of women with HIV may be vulnerable to ART‐exposure, leading to subsequent increases in skeletal fragility,^29^ particularly following the menopause transition.^30^, ^31^ This is important as traditional fracture prediction tools may underestimate fracture risk in women with HIV, an issue that requires further research.^32^

Published data overwhelmingly support an association between ART‐exposure and loss of bone mineral over 24 months.^14^, ^15^, ^33^ Similar results seen in individuals without HIV exposed to TDF as part of HIV pre‐exposure prophylaxis (PrEP) regimens, with reversal of loss after TDF discontinuation, support the hypothesis that bone changes are directly related to ART rather than factors associated with HIV infection.^11^, ^18^, ^34^, ^35^ Furthermore, improvements in BMD after switching from TDF to tenofovir alafemamide fumarate (TAF) implicate TDF as the main driver of bone loss.^36^ However, whether this is a direct effect of TDF on the skeleton or one mediated via increases in PTH remains unclear. It might represent a catabolic window, as proposed by Overton and colleagues, wherein a high bone turnover state with excess bone resorption, may be a central mechanism of ART‐associated bone loss.^37^ Such potential mechanisms, their duration, and ultimate skeletal consequences require further study.

Nref women and those with HIV who initiated ART gained weight rapidly at 12 months^5^; this continued to 24 months because of an increase in fat mass rather than lean mass. No change in weight or body composition was seen in women with HIV not exposed to ART. Rapid and sustained increases in adiposity are likely to be contributory to future cardiometabolic disease risk in women regardless of HIV status.^38^, ^39^, ^40^ Those living with HIV may be at higher risk of such outcomes,^41^, ^42^ though the precise mechanisms are unknown. There is some evidence suggesting that South Africans with HIV receive enhanced levels of care for their obesity‐related conditions compared with those without HIV.^43^ This may, in part, explain the lack of weight gain in the ART‐N group; however, there are many unanswered questions concerning the overlapping epidemic of HIV and cardiometabolic disease in Africa^44^ and how this interplay influences future fracture risk.

Unlike previous reports,^45^, ^46^, ^47^ we have no indication in this group of South African women that either HIV infection or ART exposure was associated with consistent differences in vitamin D status over time, although by 24 months ART‐unexposed women had higher mean 25(OH)D than the other groups; indeed, the HIV‐negative reference group had the lowest mean concentration at 24 months. The mean 25(OH)D concentration in all three groups exceeded 50 nmol/L at all timepoints.

The data presented in this longitudinal subset, demonstrating no significant group‐dependent changes over time in serum phosphate and TmP/GFR, argue against perturbations in renal phosphate handling as the key mechanism to explain aBMD loss with ART exposure. CRP and ferritin concentrations, both acute phase markers, were higher for the ART‐Y group at baseline than for the other groups, but not at 12 and 24 months. This likely represents improvements in inflammation associated with initially untreated advanced HIV infection,^48^ which improved with immune restoration, though other mechanisms are possible.^49^

The strengths of the study were its longitudinal design, that no participant was exposed to ART at baseline, the inclusion of an age‐appropriate HIV‐negative reference group and an ART‐unexposed group, as well as the fact that the majority of the ART‐Y group received standard South African first‐line treatment, thereby minimizing the confounding effects of various ART combinations. It is limited by several factors including its observational design, the restricted number of participants retained in the cohort to 24 months, the absence of HIV‐specific clinical data (eg, HIV‐viral load, duration of HIV infection, and adherence to ART), and the fact that not all women in the ART‐Y group were exposed uniformly to ART—the median duration of exposure by 24 months was 685 [591, 742] days.

In summary, this study suggests that in urban, black South African women, HIV infection per se has no discernible negative effects on bone mineral status over a 24‐month period, but that exposure to TDF‐based ART is associated with a loss of bone mineral and an increase in bone turnover that largely stabilizes by 24 months. Although our data suggest that the loss of aBMD plateaus after 12 months in these premenopausal women, and so provide some reassurance, we cannot predict whether there may be an exaggerated trajectory of menopausal bone loss or fracture risk. Prodrugs of TDF such as TAF have been repeatedly demonstrated to be more bone‐sparing than TDF.^33^, ^50^ However, it is unknown, at this stage, if this will affect future fracture risk.^51^ TAF is not widely available in Africa; for cost reasons, it is unlikely to replace TDF for some time in the treatment and prevention of HIV or hepatitis B. Because TDF is currently part of first‐line ART in many—particularly low‐income—countries, there is a need for future research, with longer follow‐up, to determine the long‐term skeletal effects of HIV infection, ART exposure, and other lifestyle^10^ factors to inform policies on osteoporosis and fracture prevention within an African context.

## Disclosure

The authors state that they have no conflicts of interest.

## Supporting information


**Appendix S1:** Supplementary data

**Supplementary Table S1.** Anthropometry and bone mineral densities by ART status in women participating at Baseline, 12 and 24 months
**Supplementary Table S2.** Within‐individual change from Baseline in bone mineral densities and anthropometry by ART status at 12 and 24 months
**Supplementary Table S3.** Difference in bone mineral densities and anthropometry relative to Nref in ART‐N and ART‐Y at Baseline, 12 and 24 months
**Supplementary Table S4.** Biochemistry by ART status at 24 m in women participating at Baseline, 12 and 24 months
