# Supplementary material for: Bone Mineral Density, Body Composition, and Mineral Homeostasis Over 24 Months in Urban South African Women With HIV Exposed to Antiretroviral Therapy
Source: JBMR Plus. 2020 Mar 18;4(5):e10343. doi: 10.1002/jbm4.10343 (PMC7202419; doi:10.1002/jbm4.10343)
Supplement: Supplementary file 1 — Appendix S1: Supplementary data [file JBM4-4-e10343-s001.docx]

Supplementary data. Hamill MM et al.

# Laboratory measures

Biochemical analysis was conducted at MRC Human Nutrition Research (HNR, subsequently known as the MRC Elsie Widdowson Laboratory), Cambridge, UK, using commercially available platforms and assay kits as follows: calcium, phosphate, magnesium, albumin, ferritin and CRP (Thermo Konelab 20i, Thermo Fischer Scientific, Vantaa, Finland); plasma intact PTH, β-CTX and serum P1NP (iSys, Immunodiagnostics Systems Ltd, Tyne and Wear, UK). Serum 25(OHD) assays (Liaison, DiaSorin Inc., Stillwater, MN, USA) for samples collected at baseline and 12 months were conducted at DPHRU, those collected at 24 months at HNR. The DPHRU and HNR laboratories both held a certificate of proficiency for the time of the study from the International Vitamin D External Quality Assessment Scheme (DEQAS, [www.deqas.org](http://www.deqas.org)) and cross-calibration using baseline samples showed excellent agreement between the two laboratories. The numbers of samples successfully collected, transported and analyzed varied depending on the analyte and timepoint, and are detailed in the results section (footnote to Table 2).

The following compensation equation provided by the manufacturer was applied to measured serum creatinine values to provide traceability to the international reference method (Isotope Mass Spectrometry): Compensated Creatinine (S_Cr_) (µmol/l) = (measured value-26.8)x1.168 µmol/l. Estimated glomerular filtration rate was calculated using the *Chronic Kidney Disease* Epidemiology Collaboration (CKD-EPI) formula for females [1] but without the factor for African-American ethnicity [2], as per the South African guidelines: eGFR (ml/min/1.73m^2^) **=** 141 × min (S_Cr_/0.7, 1)^-0.329^ × max(S_Cr_/0.7, 1)^-1.209^ × 0.993^age^ × 1.018, where S_Cr_ is serum creatinine concentration in mg/dl (i.e. S**_Cr_** in µmol/l x 0.0113) and age is in years. The ratio of tubular maximum reabsorption rate of phosphate to glomerular filtration rate (TmP/GFR) was derived using the following equations after calculation of the tubular resorption of phosphate (TRP):(a) if TRP ≤0.86 then TmP/GFR mmol/l = SP x TRP or (b) if TRP >0.86 then TmP/GFR mmol/l = 0.3 x SP x [TRP/(1-(0.8 x TRP))]where TRP = [(UP/SP) x (S_Cr_/U_Cr_)] and SP and S_Cr_ are the serum phosphate (mmol/l) and creatinine (mmol/l) concentrations and UP (mmol/l) and U_Cr_ (mmol/l) are their respective fasting urine concentrations.

1. Levey AS, Coresh J, Greene T, et al. Using standardized serum creatinine values in the modification of diet in renal disease study equation for estimating glomerular filtration rate. Ann Intern Med **2006**; 145(4): 247-54.

2. van Deventer HE, George JA, Paiker JE, Becker PJ, Katz IJ. Estimating glomerular filtration rate in black South Africans by use of the modification of diet in renal disease and Cockcroft-Gault equations. Clin Chem **2008**; 54(7): 1197-202.
