# Supplementary material for: Bone Mineral Density, Body Composition, and Mineral Homeostasis Over 24 Months in Urban South African Women With HIV Exposed to Antiretroviral Therapy
Source: JBMR Plus. 2020 Mar 18;4(5):e10343. doi: 10.1002/jbm4.10343 (PMC7202419; doi:10.1002/jbm4.10343)
Supplement: Supplementary file 2 — Supplementary Table S1. Anthropometry and bone mineral densities by ART status in women participating at Baseline, 12 and 24 months Supplementary Table S2. Within‐individual change from Baseline in bone mineral densities and anthropometry by ART status at 12 and 24 months Supplementary Table S3. Difference in bone mineral densities and anthropometry relative to Nref in ART‐N and ART‐Y at Baseline, 12 and 24 months Supplementary Table S4. Biochemistry by ART status at 24 m in women participating at Baseline, 12 and 24 months [file JBM4-4-e10343-s002.doc]

Supplementary Table 1. Anthropometry and bone mineral densities by ART status in women participating at Baseline, 12 and 24 months

Nref ART-N ART-Y Interaction*

Baseline 12 months 24 months Baseline 12 months 24 months Baseline 12 months 24 months *p*

*Bone mineral density (aBMD g/cm^2^)*

Lumbar spine 1.015±0.123 1.031±0.119 1.039±0.122**^l^** 1.038±0.127 1.040±0.116 1.043±0.120 1.012±0.123**^f^** 0.983±0.124**^a,d,h^** 0.990±0.127**^a,d,l^** <0.001

Total hip 1.018±0.137 1.041±0.142**^i^** 1.073±0.139**^h,j^** 1.032±0.145 1.051±0.146 1.079±0.147**^i,j^** 0.978±0.122**^a,d^** 0.985±0.126**^a,d^** 1.002±0.124**^a,d^** 0.03

Femoral neck 0.924±0.123 0.923±0.137 0.960±0.152**^i,k^** 0.966±0.151**^b^** 0.962±0.144**^c^** 0.984±0.174 0.910±0.134**^d^** 0.892±0.132**^c,d^** 0.909±0.144**^a,d^** 0.09

WBLH 0.967±0.082 0.965±0.081 0.966±0.084 0.969±0.077 0.973±0.077 0.969±0.836 0.954±0.078 0.942±0.076**^a,d,i^** 0.940±0.078**^a,d,k^** 0.03

*Anthropometry*

Weight (kg) 71.8±15.3 74.4±15.8 76.4±15.8**^j^** 74.4±14.6 74.0±13.9 73.0±13.9 65.7±17.5**^a,d^** 68.4±16.7**^a,d,h^** 69.5±15.6**^a,e,j^** 0.003

BMI (kg/m^2^) 29.1±6.3 30.2±6.6 31.1±6.7**^j^** 29.3±5.1 28.9±5.2 28.6±4.7**^c^** 25.8±6.3**^a,d^** 26.9±6.0**^a,d,h^** 27.5±5.5**^a,f,i,j^** 0.002

Fat mass, WBLH (kg) 27.9±11.3 29.8±11.6 31.0±11.4**^k^** 29.0±8.5 28.8±8.5 29.4±8.8 21.5±9.9**^a,d^** 24.1±11.2**^a,d,h^** 25.8±11.6**^a,d,i,j^** 0.001

Lean mass, WBLH (kg) 38.6±4.7 38.9±4.7 39.2±5.2 39.9±6.7**^c^** 39.7±6.4 39.8±6.2 37.5±5.2**^d^** 38.3±5.8**^e^** 38.3±5.3**^f^** 0.63

Fat:lean^2^ (1000*kg/kg^2^) 18.4±5.0 19.3±4.9 20.0±5.3**^l^** 18.3±4.1 18.6±4.7 18.7±4.6 14.8±5.0**^a,d^** 16.0±5.0**^a,d,i^** 17.2±5.3**^a,j^** 0.007

Waist (cm) 88.6±12.4 91.9±12.4 90.2±18.5**^k^** 90.5±13.8 92.7±13.4 87.5±22.5 86.7±15.3**^f^** 89.2±14.6**^f^** 86.9±19.0 0.72

Hip (cm) 109.5±12.4 111.6±13.4 114.4±13.0**^k^** 109.9±102.0 110.3±9.3 111.1±7.9 100.2±14.5**^a,d^** 105.3±12.4**^a,d,g^** 106.6±12.0**^a,f,j^** 0.002

Waist:hip (cm/cm) 0.81±0.07 0.82±0.07 0.79±0.14 0.82±0.08 0.84±0.08 0.82±0.09 0.87±0.08**^a,e^** 0.84±0.07 0.81±0.14**^l^** 0.02

**Footnote to Supplementary Table 1**

Nref, *n* = 39, HIV-negative; ART-N, *n* = 28, people with HIV (PWH) not on ART 0-24m; ART-Y, *n* = 43 PWH on ART at 24 months who initiated prior to 12 months, WBLH = whole body less head. Data are means ± SDs, aBMD data are unadjusted. *p = significance of group-by-timepoint interaction term in the 3-timepoint model (0/12/24 months).

Significance of differences from Scheffé *post hoc* tests from hierarchical linear models of the variable in natural logarithms in the 3-timepoint model with timepoint (0/12/24 months), group (Nref/ART-N/ART-Y), ID (nested within group) and a group-by-timepoint interaction, as follows:

between ART-N or ART-Y and Nref at each timepoint **^a^** ≤0.001, **^b^** ≤0.01, **^c^** ≤0.05;

between ART-N and ART-Y at each timepoint: **^d^** ≤0.001, **^e^** ≤0.01, **^f^** ≤0.05;

between 0 and 12 or between 12 and 24 months in each group: **^g^** ≤0.001, **^h^** ≤0.01, **^i^** ≤0.05.

between 0 and 24 months in each group **^j^** ≤0.001, **^k^** ≤0.01, **^l^** ≤0.05

Variables with missing datapoints for women participating at both 12 and 24 months are:

Weight, BMI, Waist circumference: at 24 months Nref = 2; ART-N = 3; ART-Y = 5

Hip circumference, waist:hip ratio: at 24 months Nref = 2; ART-N = 5; ART-Y = 5

DXA body composition variables: at baseline Nref = 0; ART-N = 0; ART-Y = 1; at 24 months Nref = 0; ART-N = 1; ART-Y = 1

DXA hip variables: at baseline Nref = 1; ART-N = 0; ART-Y = 1; at 24 months Nref = 0; ART-N = 1; ART-Y = 2

Supplementary Table 2. Within-individual change from Baseline in bone mineral densities and anthropometry by ART status at 12 and 24 months

Nref ART-N ART-Y

%∆12 months %∆24 months %∆12 months %∆24 months %∆12 months %∆24 months

*aBMD unadjusted*

Lumbar spine +1.7±0.8 +2.4±0.8**^c^** +0.4±0.9 +0.6±0.9 -2.9±0.7**^b^** -2.4±0.8**^c^**

Total hip +2.4±0.8**^c^** +5.6±0.8**^a^** +1.8±0.9 +4.7±0.9**^a^** +0.2±0.8 +2.2±0.8

Femoral neck +0.2±1.1 +3.9±1.1**^b^** -0.4±1.3 +1.5±1.3 -1.9±1.0 -0.3±1.0

WBLH -0.2±0.5 -0.2±0.5 +0.4±0.5 -0.1±0.5 -1.5±0.4**^c^** -1.6±0.4**^b^**

*aBMD adjusted*

Lumbar spine +1.4±0.8 +2.0±0.8 +0.5±0.9 +0.6±0.9 -3.1±0.8**^b^** -2.2±0.9

Total hip +2.4±0.9 +5.6±0.9**^a^** +3.1±1.1 +6.3±1.2**^a^** -0.1±0.9 +1.3±0.9

Femoral neck -0.1±1.1 +2.7±1.1 -0.3±1.2 +0.7±1.3 -2.3±1.0 -1.8±1.1

WBLH aBMD +0.2±0.5 +0.3±0.5 +0.6±0.5 +0.1±0.6 -1.0±0.5 -0.5±0.5

*Anthropometry*

Weight +3.6±1.3 +5.9±1.3**^a^** -0.4±1.5 +0.3±1.6 +4.4±1.2**^b^** +8.2±1.3**^a^**

BMI +3.6±1.3 +6.6±1.3**^a^** -1.1±1.5 +0.8±1.6 +4.4±1.2**^b^** +8.7±1.3**^a^**

Fat mass +7.3±2.9 +11.8±2.9**^b^** -0.1±3.5 +0.9±3.5 +10.7±2.9**^b^** +20.5±2.9**^a^**

Lean mass +1.0±0.8 +1.5±0.8 -0.3±1.0 +0.01±1.0 +1.1±0.8 +1.8±0.8

Fat:Lean^2^ +5.2±2.8 +8.8±2.8**^c^** +0.6±3.3 +0.9±3.3 +8.6±2.7**^c^** +16.9±2.7**^a^**

**Footnote to Supplementary Table 2**

Nref, *n* = 39, HIV-negative; ART-N, *n* = 28, people with HIV (PWH) not on ART 0-24m; ART-Y, *n* = 43; PWH on ART at 24 months who initiated prior to 12 months, WBLH = whole body less head. Data are percentage mean changes within individuals from baseline ± SEs.

Significance of within-individual changes from baseline in each group at 12 and 24 months from Scheffé *post hoc* tests hierarchical linear models of the variable in natural logarithms in the 3-timepoint model with timepoint (0/12/24 months), group (Nref/ART-N/ART-Y), ID (nested within group) and a group-by-timepoint interaction:

**^a^** ≤0.001, **^b^** ≤0.01, **^c^** ≤0.05.

The significance of comparisons between groups at each timepoint can be found in Supplementary Tables 1 and 2.

Supplementary Table 3. Difference in bone mineral densities and anthropometry relative to Nref in ART-N and ART-Y at Baseline, 12 and 24 months

ART-N ART-Y

Baseline 12 months 24 months Baseline 12 months 24 months

% % % % % %

*aBMD unadjusted*

Lumbar spine +2.2±0.8 +0.9±0.8 +0.4±0.8 -0.4±0.8 -5.0±0.8**^a^** -5.2±0.8**^a^**

Total hip +1.5±0.9 +0.9±0.9 +0.7±0.9 -3.1±0.8**^a^** -5.4±0.8**^a^** -6.5±0.8**^a^**

Femoral neck +4.5±1.2**^b^** +3.9±1.2**^c^** +2.1±1.2 -1.3±1.1 -3.4±1.1**^c^** -5.6±1.1**^a^**

WBLH +0.2±0.5 +0.8±0.5 +0.4±0.5 -1.2±0.5 -2.5±0.4**^a^** -2.6±0.5**^a^**

*aBMD adjusted*

Lumbar spine +1.4±1.1 +0.5±1.0 -0.02±1.1 -0.2±1.2 -4.6±1.0**^a^** -4.5±1.1**^b^**

Total hip +0.5±1.0 +1.2±1.0 +1.3±1.1 +0.7±0.9 -1.8±0.9 -3.6±1.0**^b^**

Femoral neck +4.1±1.2**^c^** +3.9±1.1**^c^** +2.1±1.2 +0.8±1.2 -1.4±1.1 -3.7±1.2**^c^**

WBLH -0.2±0.5 +0.2±0.5 -0.4±0.6 -1.9±0.5**^b^** -3.1±0.5**^a^** -2.7±0.5**^a^**

*Anthropometry*

Weight +3.8±1.4 -0.1±1.4 -1.8±1.5 -9.8±1.2**^a^** -9.0±1.2**^a^** -7.5±1.3**^a^**

BMI +1.0±1.4 -3.7±1.4 -4.9±1.5**^c^** -12.2±1.3**^a^** -11.5±1.3**^a^** -10.1±1.3**^a^**

Fat mass +6.3±3.2 -1.1±3.2 -4.6±3.2 -27.3±2.9**^a^** -23.8±2.9**^a^** -18.6±2.9**^a^**

Lean mass +2.8±0.9**^c^** +1.4±0.9 +1.3±0.9 -1.9±0.8 -1.8±0.8 -1.6±0.8

Fat:Lean^2^ +0.7±3.1 -3.9±3.1 -7.2±3.1 -23.5±2.8**^a^** -20.2±2.7**^a^** -15.4±2.8**^a^**

**Footnote to Table 3**

Nref, *n* = 39, HIV-negative; ART-N, *n* = 28, people with HIV (PWH) not on ART 0-24m; ART-Y, *n* = 43; PWH on ART at 24 months who initiated prior to 12 months, WBLH = whole body less head. Data are percentage mean differences relative to Nref at the same timepoint ± SEs.

Significance of differences between ART-N or ART-Y and Nref at each timepoint from Scheffé *post hoc* tests hierarchical linear models of the variable in natural logarithms in the 3-timepoint model with timepoint (0/12/24 months), group (Nref/ART-N/ART-Y), ID (nested within group) and a group-by-timepoint interaction:

**^a^** ≤0.001, **^b^** ≤0.01, **^c^** ≤0.05.

The significance of other comparisons between groups and across time can be found in Supplementary Tables 1 and 3.

Supplementary Table 4. Biochemistry by ART status at 24m in women participating at Baseline, 12 and 24 months

Nref ART-N ART-Y Interaction*

Baseline 12 months 24 months Baseline 12 months 24 months Baseline 12 months 24 months *p*

*Serum*

25(OH)D (nmol/L) 58.4±15.6 64.9±16.4 63.8±19.2 64.0±15.9 69.0±18.9 74.3±14.5**^b,l^** 62.0±22.5 60.3±20.2**^f^** 67.4±18.1 0.14

Phosphate (mmol/L) 1.06±0.16 1.15±0.20**i** 1.01±0.15**^g^** 1.13±0.21 1.15±0.19 1.03±0.18 1.13±0.19 1.21±0.24 1.03±0.14**^g,l^** 0.49

Calcium_corr_ (mmol/L) 2.49±0.10 2.48±0.09 2.45±0.10 2.52±0.13 2.48±0.09 2.46±0.09 2.51±0.12 2.47±0.11 2.44±0.09**^k^** 0.83

Magnesium (mmol/L) 0.80±0.06 0.81±0.07 0.80±0.08 0.80±0.08 0.79±0.07 0.77±0.07 0.79±0.05 0.80±0.05 0.79±0.07 0.77

Albumin (g/L) 41.8±3.3 40.6±3.3 38.9±2.8 40.9±2.6 39.0±4.1**^c^** 37.7±4.3**^c,h^** 35.4±6.2**^a,d^** 38.7±4.7**^g^** 38.9±3.5**^j^** <0.001

Creatinine# (µmol/L) 67.8±9.2 68.5±8.7 66.3±10.7 65.9±8.2 62.7±9.0**^c^** 58.7±7.4**^b^** 63.3±10.3 64.0±11.4 63.5±11.6**^f^** 0.14

eGFR (ml/min/1.73m^2^) 102.7±17.0 100.8±14.1 102.3±15.5 103.7±12.2 107.5±13.2 111.6±10.3**^b^** 106.4±14.8 105.3±14.2 104.2±14.0**^f^** 0.18

TALP^¶^ (U/L) 43.4[38.7,56.2] 52.8[42.9,61.0] 51.1[40.8,60.0] 47.4[39.4.53.7] 45.7[40.2,59.5] 47.5[38.1,58.9] 48.2[36.3,61.0] 79.3[57.7,89.3]**^a,d,g^** 67.3[54.1,83.8]**^a,d,j^** <0.001

P1NP^¶^(µg/L) nd 46.1[35.8,61.0] 46.4[36.6,62.1] nd 50.8[37.5,71.5] 49.0[36.2,57.8] nd 75.4[58.9,102.3]**^a,d^** 67.2[46.9,86.7]**^a,e,i^** -

β-CTX^¶^ (ng/L) nd 109[69,189] 151[109,263]**^h^** nd 131[86,233] 176[115,252]**^i^** nd 183[120,256]**^a,e^** 219[131,310] -

PTH^¶^(ng/L) nd 23.3[15.2,31.1] 37.9[30.2,46.5]**^g^** nd 21.1[14.0,27.4] 27.6[20.7,38.8]**^c,h^** nd 23.0[16.9,33.9]**^f^** 40.6[29.4,52.5]**^e,h^** -

CRP^¶^ (mg/L) 4.5[3.1,6.5] 4.2[3.1,7.4] 4.1[3.1,5.0] 4.3[2.9,6.2] 4.4[3.7,5.9] 4.2[3.7,5.4] 6.6[3.3,27.1]**^e^** 4.3[3.5,15.3] 5.1[3.8,13.2] 0.35

Ferritin^¶^ (µg/L) 24.1[12.1,47.7] 32.3[13.1,80.0] 44.3[13.6,77.4] 42.8[18.4,67.3] 42.2[11.8,59.3] 38.5[16.8,63.9] 51.1[27.1,120.4]**^a^** 16.7[10.7,42.4]**^g^** 25.4[15.4,30.2]**^j^** <0.001

*Urine*

Phosphate:Cre 1.01±0.40 1.00±0.53 0.99±0.62 1.24±0.58 1.28±0.69 1.18±0.64 1.24±1.39 1.41±0.64 1.28±0.69**^c^** 0.35

Calcium:Cre^¶^ 0.08[0.03,0.18] 0.08[0.03,0.15] 0.06[0.03,0.15] 0.08[0.04,0.11] 0.08[0.03,0.13] 0.11[0.06,0.12] 0.07[0.05,0.21] 0.07[0.03,0.18] 0.08[0.02,0.19] 0.53

Magnesium:Cre 0.17±0.07 0.17±0.08 0.15±0.08 0.15±0.07 0.15±0.07 0.15±0.04 0.17±0.09 0.23±0.12**f** 0.19±0.10 0.07

TmP/GFR (mmol/L) 1.13±0.27 1.26±0.32 1.08±0.26**^h^** 1.21±0.28 1.20±0.26 1.10±0.27 1.27±0.39 1.24±0.33 1.03±0.21**^h^** 0.38

**Footnote to Table 4**

Nref, *n* = 39, HIV-negative; ART-N, *n* = 28, people with HIV (PWH) not on ART 0-24m; ART-Y, *n* = 43; PWH on ART at 24 months who initiated prior to 12 months. Data are means ± SDs for normal distributions, for those with positive skew (¶) are median [25,75 percentile]. *Interaction*p* = significance of group-by-timepoint interaction term in the 3-timepoint model (0/12/24 months). #Serum creatinine values, assayed using the Jaffe method, were corrected to provide traceability to the reference method using the compensation equation: compensated creatinine (µmol/l) = (measured value-26.8)x1.168 µmol/l; nd, not determined. Abbreviations are 25(OH)D, 25-hydroxyvitamin D; eGFR, estimated glomerular filtration rate using the CKD-EPI formula with no black factor; TALP, total alkaline phosphatase; P1NP, serum type 1 procollagen N-terminal; β-CTX, serum collagen type 1 cross-linked β-C-telopeptide; serum type 1 procollagen N-terminal; PTH, parathyroid hormone; CRP, c-reactive protein; Cre, urine creatinine used to develop urine mineral ratios in mmol/mmol; TmP, tubular maximum reabsorption rate of phosphate. Baseline values for PTH, P1NP and β*-*CTX were not determined.

Significance of differences from Scheffé *post-hoc* tests from hierarchical linear models of the variable in natural logarithms in the 3-timepoint model with timepoint (0/12/24 months), group (Nref/ART-N/ART-Y), ID (nested within group) and a group-by-timepoint interaction, as follows:

between ART-N or ART-Y and Nref at each timepoint **^a^** ≤0.001, **^b^** ≤0.01, **^c^** ≤0.05

between ART-N and ARTY at each timepoint: **^d^** ≤0.001, **^e^** ≤0.01, **^f^** ≤0.05;

between 0 and 12 or between 12 and 24 months in each group: **^g^** ≤0.001, **^h^** ≤0.01, **^i^** ≤0.05.

between 0 and 24 months in each group **^j^** ≤0.001, **^k^** ≤0.01, **^l^** ≤0.05

Numbers of missing biochemical datapoints for women participating at both 12 and 24 months are:

25(OH)D, Baseline: Nref = 0, ART-N = 0, ART-Y = 0; 12 months: Nref = 2, ART-N = 0, ART-Y = 1; 24 months: Nref = 6, ART-N = 3, ART-Y = 4;

P1NP, CTX, 12 months: Nref = 1, ART-N = 0, ART-Y = 0; 24 months: Nref = 7; ART-N = 3, ART-Y = 6;

PTH, 12 months: Nref = 1; ART-N = 2 ART-Y = 3; 24 months: Nref = 7, ART-N = 3, ART-Y = 6;

Ferritin, CRP, Baseline: Nref = 11, ART-N = 7, ART-Y = 9; 12 months: Nref = 1, ART-N = 0, ART-Y = 0; 24 months: Nref = 5, ART-N = 2, ART-Y = 4;

Other blood analytes, Baseline: Nref = 12, ART-N = 10, ART-Y = 9; 12 months: Nref = 1, ART-N = 0, ART-Y = 0; 24 months: Nref = 5, ART-N = 2, ART-Y = 4;

Urine mineral/creatinine ratios, Baseline: Nref = 10, ART-N = 9, ART-Y = 18; 12 months: Nref = 4, ART-N = 3 ART-Y = 9; 24 months: Nref = 4; ART-N = 2, ART-Y = 6;

TmP/GFR, Baseline: Nref = 21, ART-N = 16, ART-Y = 22; 12 months: Nref = 5, ART-N = 3, ART-Y = 9; 24 months: Nref =9, ART-N = 5, ART-Y = 9.
